# A Flare for the Unexpected: Bone Flare as Response to Tyrosine Kinase Inhibitor Treatment in a Lung Cancer Patient: New osteoblastic bone lesions in a lung cancer patient may represent bone flare and should not be misdiagnosed as disease progression

**DOI:** 10.5334/jbsr.1907

**Published:** 2020-04-27

**Authors:** Charlotte De Bondt, Annemiek Snoeckx, Jo Raskin

**Affiliations:** 1 Antwerp University Hospital and University of Antwerp, BE

**Keywords:** lung cancer, bone flare, osteoblastic bone lesions, metastases, pseudo lesions

## Abstract

We report the case of a 72-year-old female never-smoker with stage IV endothelial growth factor receptor (EGFR) mutated lung adenocarcinoma. This patient was started on first line tyrosine kinase inhibitor (TKI) and seemingly developed new bone metastases under this treatment. As there was a remarkable discrepancy between the partial response seen in the primary tumor and non-osseous metastatic locations, the possibility of a bone flare phenomenon was considered. In this case report, we demonstrate that new bony lesions are not always synonymous with disease progression.

## Introduction

The development of bone metastases is common in non-small cell lung cancer (NSCLC) as about 20–40 percent of patients will be diagnosed with metastatic bone lesions at some point during the course of their disease [1]. In the majority of cases these lesions are osteolytic, but osteoblastic bone metastases in NSCLC have been reported, primarily in adenocarcinoma [2,3]. The finding of new metastatic (bone) lesions will generally prompt a change of treatment.

## Case report

**Figure 1:**
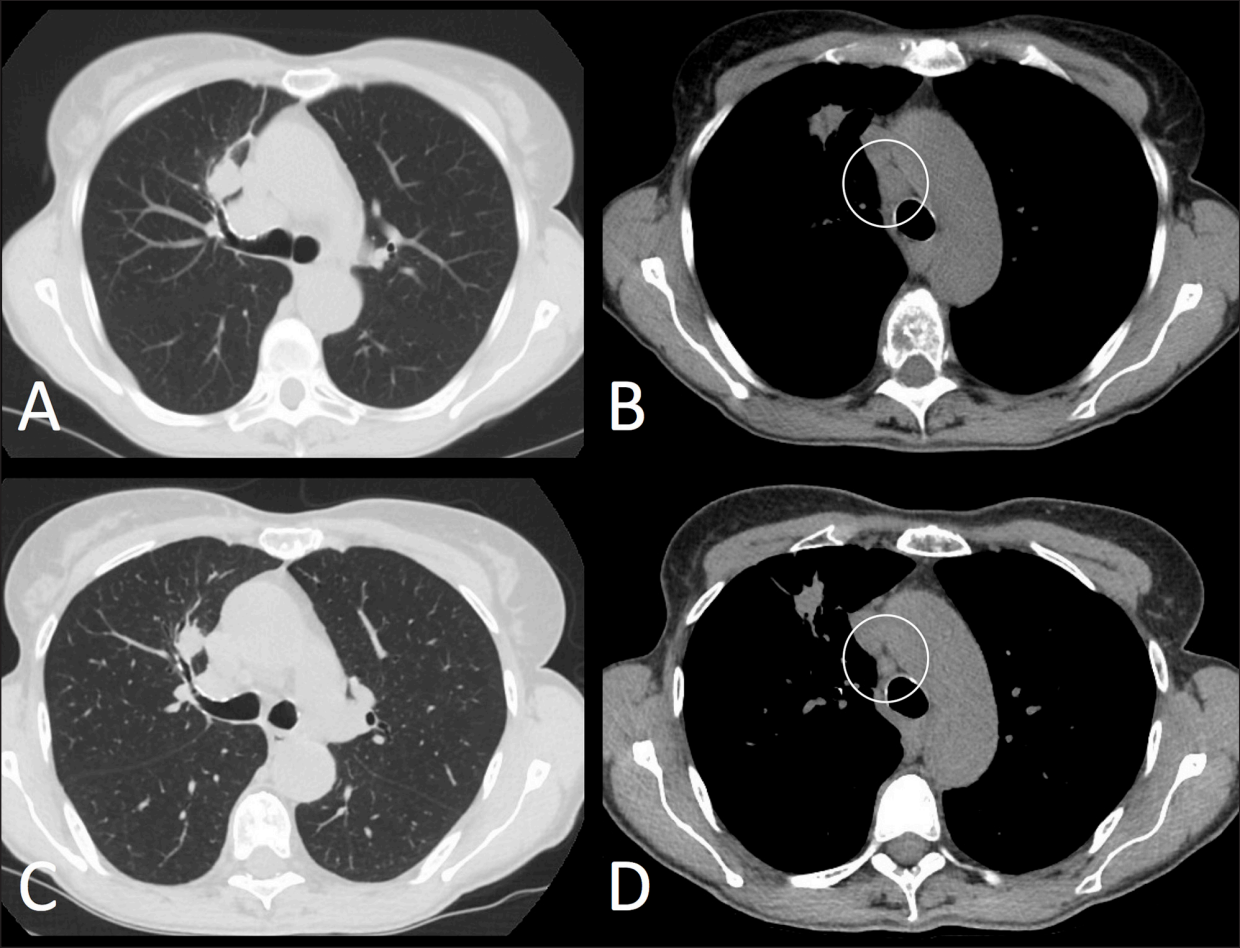
AB = baseline, CD = eight weeks follow-up. Axial non-contrast enhanced CT-images in lung and mediastinal window settings show a decrease in size of the primary lung tumor in the right upper lobe **(A, C)**. Also note the decreased short axis of the mediastinal adenopathies **(B, D)**. Regarding the extra-osseous lesions, patient would have been classified according to RECIST 1.1. as partial response.

**Figure 2:**
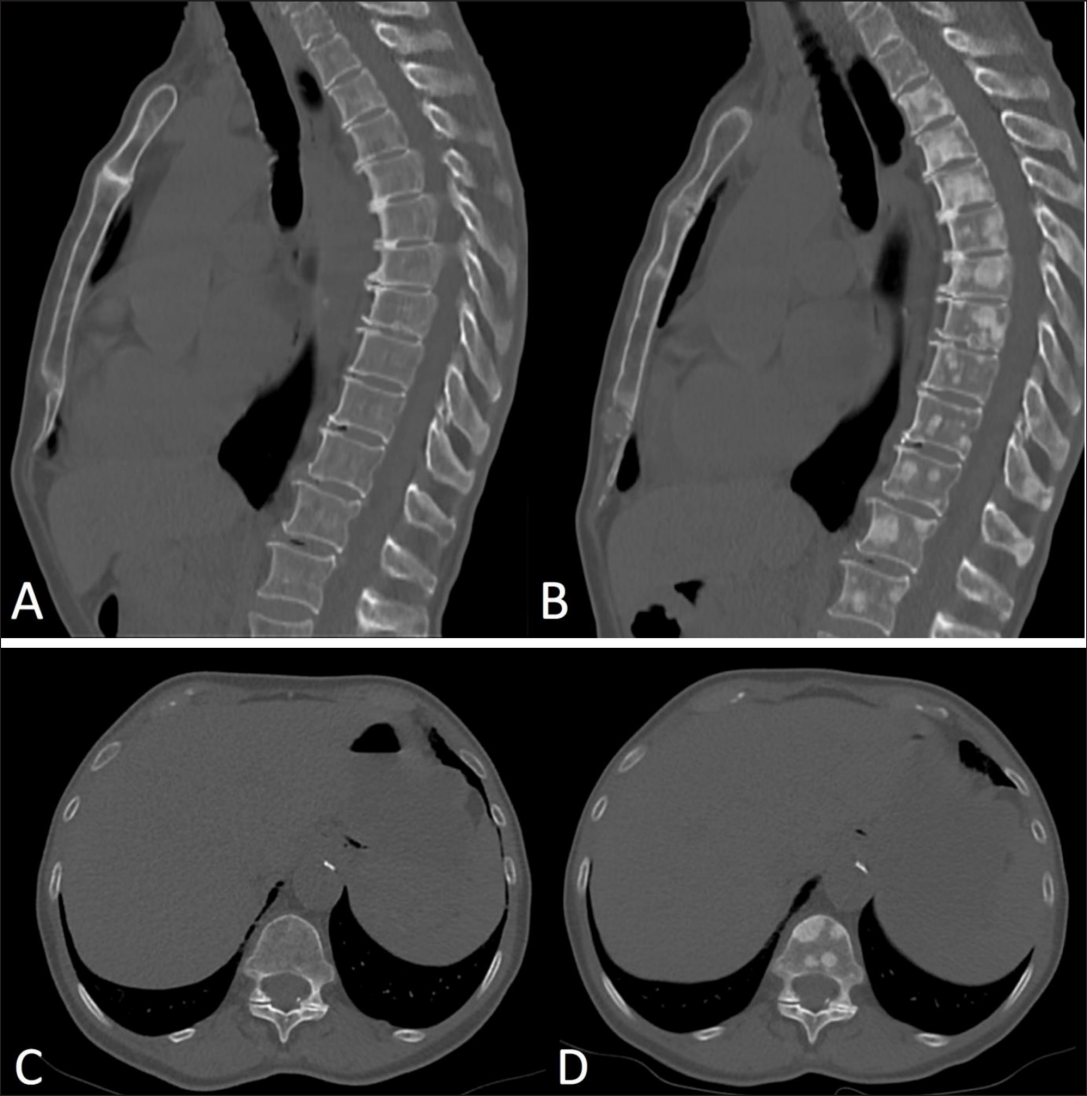
Sagittal and axial CT-images in bone window setting (Figure 1). The baseline study **(A, C)** shows no focal lytic or blastic bone lesions. First follow-up CT after eight weeks of treatment with erlotinib **(B, D)** shows numerous new blastic bone lesions in the spine, ribs and sternum. Misinterpretation of these findings as new metastases would classify this patient as progressive cancer disease.

A 72-year-old female never-smoker was diagnosed with stage IV adenocarcinoma of the lung. The primary lesion was located in the right upper lobe and measured 2.9 cm in its greatest diameter. Staging was performed using computed tomography (CT) of chest and abdomen as well as a 18-F-Fluorodeoxyglucose Positron Emission Tomography-CT (PET-CT). These examinations showed affected hilar, mediastinal and supraclavicular lymph nodes and distant metastases in liver and bone marrow (TNM 7: cT1bN3M1b). There was no evidence on CT of focal lytic or blastic bone lesions. Because next-generation sequencing (NGS) revealed an activating EGFR mutation in exon 21, she was started on daily erlotinib 150 mg, an oral targeted EGFR TKI. This therapy was well tolerated. On her first follow-up three months into treatment, CT showed shrinking of the primary tumor, mediastinal lymph nodes and liver metastases (Figure 1). Surprisingly a large number of new osteoblastic bone lesions were found (Figure 2).

The presence of the following key features led us to interpret these findings as an osteoblastic flaring as opposed to true disease progression. Firstly, the discrepancy in evolution between the existing tumor sites – which were regressing in response to treatment – and the development of new skeletal lesions. Secondly, the osteoblastic nature of the bony lesions as opposed to the usual osteolytic bone destruction common in lung cancer. Thirdly, there was no clinical deterioration suggestive of disease progression. Last but not least, the presence of numerous foci of high uptake in the bone marrow on the initial PET examination indicated the presence of diffuse bone marrow involvement. Consequently, treatment with erlotinib continued without interruption. Follow-up CTs were performed with two month intervals. The patient maintained a partial response until seven months into treatment, when she was admitted because of fever of unknown origin. 18F-FDG-PET revealed disease progression in lung, liver and bone, with numerous osseous lesions showing high uptake and therefore being metabolic active. A liver biopsy was performed to screen for escape mutations. In the meantime, erlotinib was paused and because of progressive deterioration she was started on carboplatin-pemetrexed. Pathology revealed a new EGFR mutation in exon 20, namely T790M, which is responsible for the acquired resistance to EGFR TKIs. This finding enables treatment with third generation EGFR TKIs, for example, osimertinib. Unfortunately, the overall physical condition of our patient no longer allowed chemotherapy or targeted therapy. She died eight months after diagnosis.

## Discussion

Osteoblastic bone flaring is a phenomenon whereby new or more prominent osteoblastic bony lesions arise in the presence of a clear therapeutic response in other tumor sites. It is caused by increased osteoblastic activity, representing healing of the bone metastases. As a result it can be considered as a sign of therapeutic efficacy [4]. It is impossible to differentiate between disease progression and osteoblastic flaring on CT scan or bone scintigraphy. CT will show osteoblastic lesions and bone scintigraphy will reveal increased osteoblastic activity.

The flare phenomenon is fairly common in patients with breast and prostate cancer undergoing systemic treatment with hormonal agents or chemotherapy [5]. As a result the Prostate Cancer Clinical Trials Working Group recommends performing a follow-up bone scintigraphy at least six weeks after the first bone scan at 12 weeks of treatment, whenever osteoblastic flaring is suspected [6]. Recently, a few cases have been reported in patients with NSCLC undergoing treatment with EGFR TKI [7,8,9]. There has also been one report of osteoblastic flaring in a NSCLC patient harboring an ALK-mutation [10].

The mechanism of osteoblastic flaring in patients with NSLCL is not fully understood. It could be a direct effect of the EGFR TKI on bone metabolism or an innate healing reaction when disease progression has been halted by successful treatment. There is in vitro evidence that the EGFR pathway stimulates formation of osteoclasts, resulting in bone resorption [11]. Inhibition of this pathway by an EGFR TKI could therefore cause activation of osteoblasts and bone formation. In contrast, Zhang et al. reviewed animal studies that showed that EGFR has an anabolic effect on bone metabolism resulting in osteoblastic activity. These authors suggest that this discrepancy may be the result of a different level of expression of EGF, for example, abnormally high in an in vitro setting matching the levels found in certain EGF-expressing tumors versus physiologic levels in animal studies [12].

Activated EGFR mutations in NSCLCs have been linked to abnormal activation of the WNT signal transduction pathway [13]. This signaling cascade plays a vital role in embryonic development and faulty activation of this cascade contributes to tumor development and growth [14]. This pathway has also been identified as being a promoter of osteoblastic activity in prostate cancer. As such this could signify another way in which EGFR mutations lead to osteoblastic bone lesions in NSCLC. Why some patients develop osteoblastic flaring and others do not is unclear.

The introduction of driver mutations and targeted therapies has profoundly changed the treatment of NSCLC. Currently targeted therapy exists for mutations or alterations in EGFR, BRAF, ERBB2, MET, ALK, ROS1 and TRK genes [15]. Further research is ongoing, and it is expected additional targetable mutations will be identified [16]. As these targeted therapies are increasingly being used, the likelihood of encountering a bone flare phenomenon is growing [7,10].

## Conclusion

The osteoblastic flare phenomenon is well-known in certain kinds of other solid tumors and has recently been reported in patients with NSCLC, specifically those treated with (EGFR) TKI. As these targeted therapies are becoming more common practice, the possibility of osteoblastic flaring should be considered to avoid a misinterpretation of radiologic findings leading to a premature cessation of a successful treatment. Timely follow-up imaging and a critical analysis of both clinical and radiological evolution are therefore vital for making the right therapeutic decisions.
