# Correction: A Flare for the Unexpected: Bone Flare as Response to Tyrosine Kinase Inhibitor Treatment in a Lung Cancer Patient

**DOI:** 10.5334/jbsr.2163

**Published:** 2020-06-08

**Authors:** Charlotte De Bondt, Annemiek Snoeckx, Jo Raskin

**Affiliations:** 1 Antwerp University Hospital and University of Antwerp, BE

**Keywords:** lung cancer, bone flare, osteoblastic bone lesions, metastases, pseudo lesions

## Abstract

This article details a correction to the article: De Bondt C, Snoeckx A, Raskin J. A Flare for the Unexpected: Bone Flare as Response to Tyrosine Kinase Inhibitor Treatment in a Lung Cancer Patient: New osteoblastic bone lesions in a lung cancer patient may represent bone flare and should not be misdiagnosed as disease progression. Journal of the Belgian Society of Radiology. 2020; 104(1):18. DOI: http://doi.org/10.5334/jbsr.1907

The article, ‘A Flare for the Unexpected: Bone Flare as Response to Tyrosine Kinase Inhibitor Treatment in a Lung Cancer Patient: New osteoblastic bone lesions in a lung cancer patient may represent bone flare and should not be misdiagnosed as disease progression’ [1] was published with an incorrect title. The correct title is: A Flare for the Unexpected: Bone Flare as Response to Tyrosine Kinase Inhibitor Treatment in a Lung Cancer Patient.

## References

[1] De Bondt C, Snoeckx A, Raskin J. A Flare for the Unexpected: Bone Flare as Response to Tyrosine Kinase Inhibitor Treatment in a Lung Cancer Patient: New osteoblastic bone lesions in a lung cancer patient may represent bone flare and should not be misdiagnosed as disease progression. Journal of the Belgian Society of Radiology. 2020; 104(1): 18 DOI: 10.5334/jbsr.1907 32377620 PMC7193755

